# Understanding and explaining differences across minds in social interaction: insights from social neuroscience and clinical psychiatry

**DOI:** 10.1007/s00406-025-02136-3

**Published:** 2025-10-30

**Authors:** Daniel Kamp, Dimitrios Bolis, Leonhard Schilbach

**Affiliations:** 1 Department of General Psychiatry 2, LVR-Klinikum Düsseldorf, Bergische Landstr. 2, Düsseldorf, 40629 Germany; 2 Laboratory for Autism and Neurodevelopmental Disorders, Center for Neuroscience and Cognitive Systems, Instituto Italiano di Tecnologia Rovereto, Palazzo Fedrigotti Corso Bettini 31, Rovereto, TN 38068 Italy; 3 Department of Psychiatry and Psychotherapy, LMU University Hospital, Ludwig-Maximilians- University Munich, Nussbaumstraße 7, Munich, 80336 Germany

Autism spectrum disorder is typically characterized by impairments of social interaction and communication as well as stereotyped, repetitive behaviors. These social impairments have traditionally been linked to deficits in ‘theory of mind’ or ‘empathy’. On the other hand, the neurodiversity movement has pointed out that differences in social cognitive abilities and potentially underlying neurobiology should not be labeled per se as deficits. Extending this idea, Damian Milton [1] introduced the notion of a ‘double empathy problem’, which suggests that social impairments associated with autism should not be solely attributed to empathy deficits on the part of an autistic interaction partner, but should be viewed as difficulties on both sides to understand the respective other. In other words, the idea of a double empathy problem suggests that while autistic persons may struggle to understand non-autistic persons, the opposite is also true leading to empathy problems on both sides. Interestingly, traditional research in the field of autism has not often discussed or systematically researched the latter case, because all attention and efforts were directed towards understanding autistic deficits [2].

The notion of a ‘double empathy problem’ resonates with what has been described as the ‘interactive turn’ in social neuroscience. Social neuroscience is a by now well-established field of research that started out with the idea of studying the neural bases of social interaction. Still, delivering on this promise or proposition has been challenging, as studying two (or even multiple) brains in real-time interaction poses numerous and profound challenges in terms of conceptualization, experimentation, methodology and data analysis. Recent reviews have demonstrated, however, that the field has been successful in meeting and (at least in part) overcoming these challenges to yield completely new insights in the workings of our social brains in direct social interaction [3]. Research has, for instance, shown that social interactions rely on both behavioral and neural synchrony across interacting brains. It has been suggested that the act of keeping together in time with others may foster our social bonds. Through coordinated behavior, individuals can merge into a coherent social entity, thereby attaining joint objectives that would remain the reach of any single member. Interestingly, behavioral coordination between people also influences how well they can make sense of each other’s minds: physically moving together with others has been shown to increase mental state attribution [4].

And, indeed, interpersonal synchrony does not stop at the level of behavior or subjective experience, but is also observable at the level of the brain. In fact, there is a growing body of literature that demonstrates brain-to-brain coupling across interacting dyads such as romantic couples, in a classroom setting and elsewhere and shows that such neural coupling goes beyond task- or stimulus-driven effects. In other words, neural synchrony is not a by-product of receiving the same sensory input, but appears to be relevant in terms of social information processing and communication [5].

In our own work, we have consequently suggested to provide a neurobiologically-oriented, integrative framework for social interactions that relies on the predictive coding account and has been referred to as the *social interaction mismatch* or *dialectical misattunement hypothesis*^*2*^. Rather than focusing on individuals or individual difficulties in social understanding, this proposal suggests to view misunderstanding as an interactional problem that should be investigated by focusing on the dynamics of social interaction and by focusing on how individual brains can process and predict social exchange, but also how interpersonal similarity across brains may account for communication success. In other words, the *dialectical misattunement hypothesis* foregrounds matches and mismatches in social interaction rather than mere function and misfunction in individual brains, thereby reframing psychopathology in terms of not only intrapersonal but also interpersonal dynamics. From this perspective, the key to understanding social dysfunction lies not in isolated neural mechanisms, but in the multiscale coordination of brains, minds and bodies in interaction, which in the field of precision medicine and clinical psychiatry might translate into the idea of an ‘inter-personalized psychiatry’.

In line with this account, behavioral research has shown that interpersonal difference values of autistic traits are more closely linked to friendship quality than autistic traits per se [6] and that higher behavioral synchrony is observed among autistic pairs than mixed dyads [7]. In line with this, Feng et al. [8] have recently shown that also neural synchronization across brains depends on the similarity in autistic traits across individuals and that groups with high or low autistic traits rely on different brain regions for synchronization amongst each other. Furthermore, a study by Bilek et al. [9] has investigated whether neural synchrony plays a causal role through which brains influence each other: Analyses of effective connectivity in dual-brain recordings were, indeed, able to demonstrate such between-brain influences that were specific to social exchange and directed from the sender’s to the receiver’s brain. In other words, a causal connection was present between the two brains and necessary to explain the data. Importantly, the conceptual framework used by Bilek et al. presupposes a structural similarity between the coupled systems, i.e. brains, which also seems consistent with the idea of interpersonal similarity as an important component of communication success.

Finally, we can ask whether a ‘double empathy problem’ really only applies to autism? As previously suggested, we see social impairments and communication difficulties as a fundamental, transdiagnostic aspect of all psychiatric disorders. While this has been increasingly recognized the underlying behavioral and neural mechanisms are still not well understood. In line with the above described research efforts, recent studies have started investigating behavioral and neural synchrony across various psychiatric disorders. Here, emerging evidence demonstrates that psychiatric disorders are, indeed, associated with lower levels of interpersonal synchronization. Consequently, restoring synchrony across patient and therapist would appear to be a potentially important goal for treatment. In concordance with this, a ground-breaking study by Bilek and colleagues [10] has demonstrated that lower neural coupling in patient-control dyads is restored when the patient no longer meets the diagnostic criteria at the end of treatment, which suggests that measures of neural synchrony could serve as a state-associated marker of disease.

Ending on a clinical note, we would like to point out that our ‘outpatient clinic for disorders of social interaction’ is now running in its tenth year and continues to attract large numbers of persons who report social interaction difficulties, relational problems and severely reduced quality of life. Importantly, a substantial proportion of these individuals, indeed, meet the diagnostic criteria of a psychiatric disorder underscoring that these social impairments represent an important and still unmet medical need that warrants both therapeutic and scientific attention within the field of psychiatry.
